# Mealworm Ethanol Extract Enhances Myogenic Differentiation and Alleviates Dexamethasone-Induced Muscle Atrophy in C2C12 Cells

**DOI:** 10.3390/life13010058

**Published:** 2022-12-24

**Authors:** Ra-Yeong Choi, Bong Sun Kim, Eu-Jin Ban, Minchul Seo, Joon Ha Lee, In-Woo Kim

**Affiliations:** Department of Agricultural Biology, National Institute of Agricultural Sciences, Rural Development Administration, Wanju 55365, Republic of Korea

**Keywords:** C2C12 cells, dexamethasone, muscle atrophy, myoblast differentiation, sarcopenia, *Tenebrio molitor* larvae

## Abstract

Aging, and other disease-related muscle disorders are serious health problems. Dexamethasone (DEX), a synthetic glucocorticoid, can trigger skeletal muscle atrophy. This study examined the effects of mealworm (*Tenebrio molitor* larva) ethanol extract (TME) on C2C12 myoblast differentiation and DEX-induced myotube atrophy. TME induced myotube formation compared to the differentiation medium (DM) group. TME also significantly increased the mRNA expression of muscle creatine kinase (*CKm*) and myogenic regulatory factors (MRFs), such as myogenin (*MyoG*), myogenic factor (*Myf*)5, and MRF4 (*Myf6*). TME dramatically increased the muscle-specific protein, MyoG, compared to the control, whereas the expression of myogenic differentiation 1 (MyoD) remained unchanged. It also activated the mammalian target of rapamycin (mTOR) signaling pathway. In the DEX-induced muscle atrophy C2C12 model, TME reduced the gene expression of *atrogin-1*, muscle RING finger protein-1 (*MuRF-1*), and *myostatin*, which are involved in protein degradation in skeletal muscles. Furthermore, TME elevated the phosphorylation of forkhead box O3 (FoxO3α) and protein kinase B (Akt). These findings suggest that TME can enhance myotube hypertrophy by regulating the mTOR signaling pathway, and can rescue DEX-induced muscle atrophy by alleviating atrophic muscle markers mediated by Akt activation. Thus, TME can be a potential therapeutic agent for treating muscle weakness and atrophy.

## 1. Introduction

Sarcopenia is a common condition in older adults characterized by progressive loss of skeletal muscle mass and strength [1]. Although aging tends to be the dominant factor, other risk factors have been found to be associated with sarcopenia such as physical inactivity, fasting, malnutrition, various systemic diseases (cancer, diabetes, renal failure, cardiac failure, chronic obstructive pulmonary disease, sepsis, burns, and trauma), mitochondrial dysfunction, apoptosis, androgen loss, decreased growth hormone levels, and insulin resistance [2]. Endogenous glucocorticoids are known to cause muscle atrophy in many pathological conditions such as cancer, burns, and sepsis by inhibiting insulin signaling [3]. One study has suggested that glucocorticoid-stimulated muscle atrophy can be caused by ubiquitin-proteasome-dependent proteolysis, although calcium-dependent protein proteolysis may also be involved [4]. Ubiquitin binds to the substrate through the sequential action of three types of proteins: E1 (ubiquitin-activating enzyme), E2 (ubiquitin-carrier or conjugating enzyme), and E3 (ubiquitin ligase), and induces protein degradation within the proteasome [5]. The two E3 ubiquitin ligases, namely muscle atrophy F-box protein (MAFbx)/atrogin-1 and muscle RING finger protein-1 (MuRF-1), are essential regulators of ubiquitin-mediated protein degradation in skeletal muscles [6]. MuRF1 was shown to degrade myosin heavy chain protein (MyHC) in dexamethasone (DEX)-treated differentiated myotubes [7]. The mammalian target of rapamycin (mTOR) is a serine/threonine kinase that plays a vital role in skeletal protein synthesis [8].

Mealworm (*Tenebrio molitor* larva), an edible insect, has attracted interest as a potential protein supplement because of its high content of beneficial nutrients, including essential amino acids, polyunsaturated fatty acids, trace elements, and vitamins [9]. Kim et al. [10] have reported that mealworm protein hydrolysate prepared with alcalase and flavorzyme is easily digested and absorbed, and it has potential as a protein source for preventing sarcopenia. Lee et al. [11] have reported that mealworm-derived protein supplementation attenuates skeletal muscle mass loss by stimulating muscle protein synthesis factors, and inhibiting muscle protein degradation factors in casting-induced hindlimb-immobilized rats. However, this effect was only evaluated in the soleus muscle, a slow-twitch fiber. Furthermore, the efficacy of mealworms in promoting myogenic differentiation and preventing glucocorticoid-induced muscle atrophy has not yet been demonstrated. Therefore, the effects of mealworm ethanol extract (TME) on the promotion of myoblast differentiation and muscle atrophy caused by DEX were examined using C2C12 cells.

## 2. Materials and Methods

### 2.1. Preparation of the Mealworm Ethanol Extract

Mealworms were washed, sterilized, freeze-dried, and pulverized using a sway-type pulverizer (KSP-35, Korea medi Co., LTD, Daegu, Korea). The freeze-dried powder was mixed with 70% ethanol, sonicated (350 J, 10 s, twice) using an ultrasonic processor (VCX500, Sonics & Materials, Inc., Newtown, CT, USA), and kept at 25 °C for 30 min. The extract was then centrifuged at 3500 rpm for 10 min. This was followed by the supernatant being separated and collected. The supernatant was filtered using a 0.45 μm PVDF syringe filter (GE Healthcare, Little Chalfont, UK) before being transferred into pre-weighed containers. The filtrate was then concentrated using a vacuum rotary evaporator (CVE-3100; EYELA, Tokyo, Japan). TME was stored at −70 °C until further use.

### 2.2. Cell Culture and Cell Differentiation

C2C12 skeletal muscle myoblasts were purchased from the American Type Culture Collection (ATCC; Manassas, VA, USA), and cultured in a growth medium (GM) consisting of Dulbecco’s modified Eagle’s medium (DMEM; HyClone Laboratories, Inc., Logan, UT, USA), 10% fetal bovine serum (FBS; Gibco, Thermo Fisher Scientific, Inc., Waltham, MA, USA), and 1% antibiotics (Gibco, Thermo Fisher Scientific, Inc., Waltham, MA, USA). C2C12 myoblasts were seeded at 1 × 10^4^ cells/well in 96-well plates or at 2 × 10^5^ cells/well in 6-well plates, and maintained in the GM until they reached confluence, in a humidified 5% CO_2_ incubator at 37 °C. To induce C2C12 myoblast differentiation, the GM was replaced with DMEM containing 2% horse serum (Gibco, Thermo Fisher Scientific, Inc., Waltham, MA, USA; differentiation medium, DM). Cells were treated with DM containing various concentrations of TME (0, 50, 100, 250, and 500 μg/mL) for 2 days and then subjected to analytical experiments. For the DEX-induced muscle atrophy study, differentiated myotubes on day 4 were pretreated with TME (0, 50, 100, 250, and 500 μg/mL) for 1 h and then treated with or without 100 μM DEX (Sigma-Aldrich, St. Louis, MO, USA) for 24 h. Subsequently, myotubes were viewed using an optical microscope (Leica DMI6000B, Leica Microsystems, Wetzlar, Germany) at 200× magnifications. The average diameters of 90 myotubes were determined from three different locations per myotube and analyzed using Image J software (National Institute of Health, Frederick, MD, USA).

### 2.3. Cell Viability

C2C12 myoblasts were seeded at 1 × 10^4^ cells/well in 96-well plates and cultured in GM for 24 h. C2C12 myoblasts were treated with TME (0, 50, 100, 250, 500 μg/mL) placed in the GM for 24 h. To study DEX-induced muscle atrophy, C2C12 myoblasts were differentiated for 4 days, followed by treatment with DEX (100 μM) along with TME (0, 50, 100, 250, 500 μg/mL) for an additional 24 h. Cell viability was measured using a CellTiter 96 AQueous One Solution Cell Proliferation Assay kit (MTS; Promega Corporation, Madison, WI, USA) according to the manufacturer’s recommendations.

### 2.4. Western Blot Analysis 

Following treatment, the cells were harvested and washed with cold phosphate-buffered saline (PBS; Caisson Labs, Inc., Smithfield, UT, USA) and lysed in M-PER mammalian protein extraction reagent containing protease and phosphatase inhibitor cocktail (Thermo Fisher Scientific, Inc., Waltham, MA, USA). The supernatant was obtained by centrifugation at 12,000 rpm for 15 min at 4 °C. Protein concentrations were determined using the bicinchoninic acid (BCA) Protein Assay Kit (Thermo Fisher Scientific, Inc., Waltham, MA, USA). Proteins were separated on 4–12% polyacrylamide gel (Invitrogen, Thermo Fisher Scientific, Inc., Waltham, MA, USA) and transferred electrophoretically to polyvinylidene difluoride membranes (Invitrogen). The membranes were blocked with 5% skim milk in Tris-buffered saline with Tween 20 (TBST; Thermo Fisher Scientific, Inc., Waltham, MA, USA) at RT for 1 h and incubated overnight at 4 °C with primary antibodies against phospho-mTOR (#2971; Cell Signaling Technology, Danvers, MA, USA), mTOR (#2972), phospho-p70 S6 kinase (#9205), p70 S6 kinase (#9202), phospho-4E-BP1 (#2855), 4E-BP1 (#9452), phospho-Akt (#4060), Akt (#4691), phospho-FoxO3α (#9466), FoxO3α (#2497), β-actin (#4967), MyoD (sc-32758; Santa Cruz Biotechnology, Dallas, TX, USA), myogenin (sc-12732), Fbx32 (ab168372; Abcam, Cambridge, UK), and MURF-1 (ab183094). The membranes were incubated with appropriate horse-radish peroxidase (HRP)-conjugated secondary antibodies (#7074 or #7076) at RT for 1 h. The target protein signals were detected using an enhanced chemiluminescence (ECL) substrate (Thermo Fisher Scientific, Inc., Waltham, MA, USA) and a chemiluminescence imaging system (Alliance Q9 advanced, Uvitec Ltd., Cambridge, UK).

### 2.5. RNA Isolation and Quantitative Real-Time PCR Analysis

Total RNA was isolated using TRIzol reagent (Invitrogen, Thermo Fisher Scientific, Inc., Waltham, MA, USA) according to the manufacturer’s specifications. RNA purity and quantity were evaluated using a Varioskan^TM^ LUX multimode microplate reader (Thermo Fisher Scientific, Inc., Waltham, MA, USA). The cDNA was generated using a high-capacity cDNA reverse transcription kit (Applied Biosystems, Thermo Fisher Scientific, Inc., Waltham, MA, USA). Quantitative real-time PCR analysis was performed using Power SYBR Green PCR master mix (Applied Biosystems, Thermo Fisher Scientific, Inc., Waltham, MA, USA) and QuantStudio 3 Real-Time PCR Instrument (Applied Biosystems, Thermo Fisher Scientific, Inc., Waltham, MA, USA). The primer sequences are shown in Table 1. The relative mRNA levels were normalized to glyceraldehyde 3-phosphate dehydrogenase (*GAPDH*) expression levels in the same samples.

### 2.6. Statistical Analysis

All data are presented as means ± standard error (SE). Statistically significant differences among groups were determined by one-way analysis of variance (ANOVA), followed by Tukey’s post hoc test using the Statistical Package for the Social Sciences (SPSS version 20, SPSS Inc., Chicago, IL, USA). Differences were considered statistically significant at *P* < 0.05.

## 3. Results

### 3.1. Effects of TME on Cell Viability of C2C12 Myoblast, and Morphology of C2C12 Myotubes

**Figure 1:**
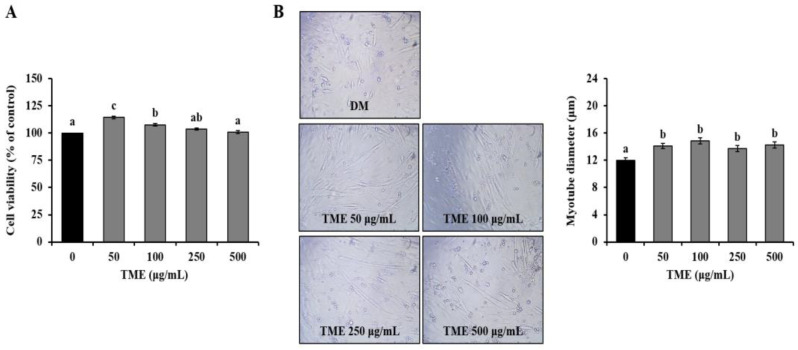
Effects of *Tenebrio molitor* larvae ethanol extract (TME) on cell viability in C2C12 myoblast and morphological alterations in C2C12 myotubes. (**A**) Cell viability of C2C12 myoblast cells in the presence of 0, 50, 100, 250, and 500 μg/mL TME for 24 h. (**B**) Representative morphological images and diameter of C2C12 myotube cells in the presence of 0, 50, 100, 250, and 500 μg/mL TME on day 2 of differentiation. The data were based on triplicate experiments. Values are expressed as means ± SE. Values not sharing a common letter (a, b, c) are significantly different among groups. Images captured at ×200 magnification. DM: differentiation medium.

We first measured the viability of C2C12 myoblasts using the MTS assay. Cell proliferation was significantly increased in the myoblasts treated with 50 and 100 μg/mL TME compared with untreated cells, and no significant difference was observed between the 500 μg/mL TME and non-treated cells (Figure 1A). Therefore, 50, 100, 250, and 500 μg/mL TME were used in further experiments. We then performed a microscopic analysis of myoblast differentiation into myotubes in the absence or presence of the TME. The TME treatment resulted in elongated and thickened cylindrical cell morphology relative to the DM group and was dose-dependent (Figure 1B).

### 3.2. Effects of TME on the Expression of Myogenic Differentiation-Related Factors in C2C12 Myotubes

**Figure 2:**
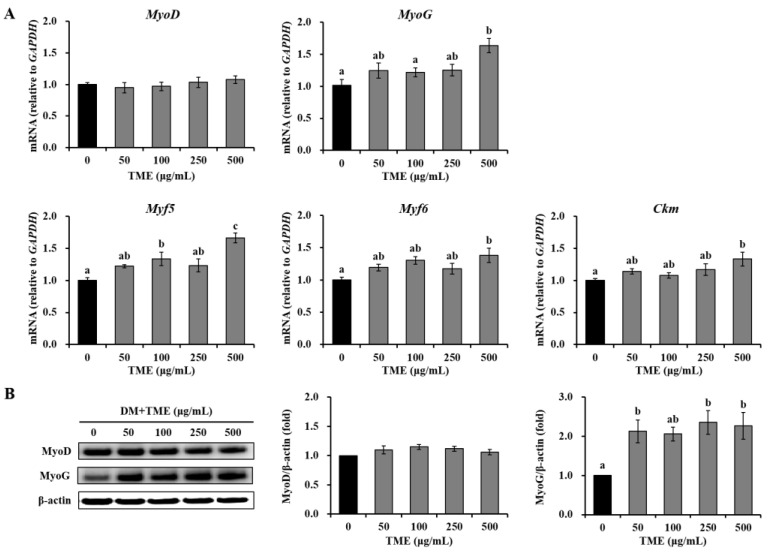
Effects of *Tenebrio molitor* larvae ethanol extract (TME) on the gene and protein expression of myogenic regulatory factors in C2C12 myotubes. C2C12 cells were treated with 0, 50, 100, 250, and 500 μg/mL TME for 2 days of differentiation. (**A**) Glyceraldehyde-3-phosphate dehydrogenase (GAPDH) was used as an internal reference gene. (**B**) β-actin was used as an internal standard. The protein expression was presented as fold-change relative to the DM group. The data were based on triplicate experiments. Values are expressed as means ± SE. Values not sharing a common letter (a, b, c) are significantly different among groups. DM: differentiation medium; *MyoD*: myogenic differentiation 1; *MyoG*: myogenin; *Myf5*: myogenic factor 5; *Myf6*: myogenic factor 6; *Ckm*: muscle creatine kinase.

To investigate the effects of TME on muscle regeneration, we evaluated the mRNA and protein expression of myogenic differentiation-related markers in C2C12 myotubes on day 2 after switching to DM. TME treatment at a high concentration (500 μg/mL) significantly increased myogenin (*MyoG*), myogenic factor (*Myf*) 5, *Myf6*, and muscle creatine kinase (*Ckm*) mRNA expression by 60.90, 65.80, 37.41, and 33.16%, respectively, compared with the DM group, and 50–250 μg/mL TME showed a slight increase (Figure 2A). There was no significant difference in myogenic differentiation 1 (*MyoD*) expression among the groups. As expected, no significant changes were observed in MyoD protein expression among the groups (Figure 2B). TME treatment (50–500 μg/mL) markedly increased MyoG protein expression by approximately 2-fold compared to that in untreated cells. These results indicate that the TME can promote the differentiation of C2C12 myoblasts.

### 3.3. Effects of TME on the mTOR Pathway in C2C12 Myotubes

**Figure 3:**
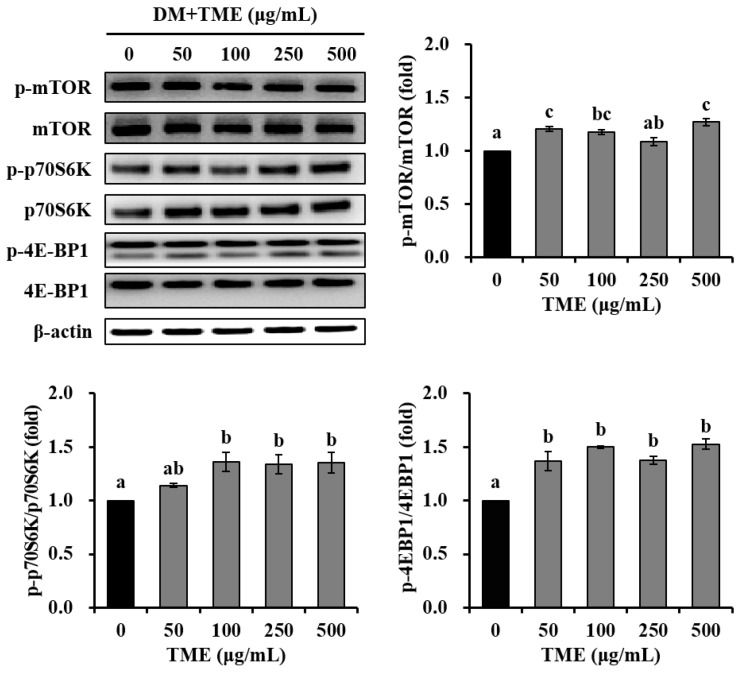
Effects of *Tenebrio molitor* larvae ethanol extract (TME) on the expression of key proteins involved in the mTOR signaling pathway in C2C12 myotubes. C2C12 cells were treated to 0, 50, 100, 250, and 500 μg/mL TME for 2 days of differentiation. β-actin was used as an internal standard. The phosphorylation protein levels were normalized to levels of total proteins, and are presented as fold-change relative to the DM group. The data were based on triplicate experiments. Values are expressed as means ± SE. Values not sharing a common letter (a, b, c) are significantly different among groups. DM: differentiation medium; mTOR: mammalian target of rapamycin; p70S6K: 70-kDa ribosomal protein S6 kinase; 4E-BP1: eukaryotic translation initiation factor 4E-binding protein 1.

Subsequently, we determined the regulatory effects of the TME on the mTOR pathway in C2C12 myotubes. TME treatment (100–500 μg/mL) significantly enhanced the phosphorylation of mTOR in differentiated C2C12 cells (Figure 3). The TME treatment effectively activated the phosphorylation of 70-kDa ribosomal protein S6 kinase (p70S6K) and eukaryotic translation initiation factor 4E (eIF4E)-binding protein 1 (4E-BP1), which are critical downstream targets of the mTOR signaling cascade. 

### 3.4. Effects of TME on Dexamethasone-Induced Cell Viability Decrease and Morphological Changes

**Figure 4:**
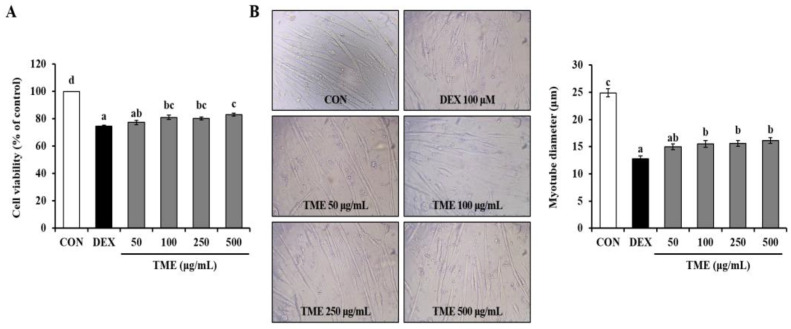
Effects of *Tenebrio molitor* larvae ethanol (TME) extract on cell viability (**A**) and myotube diameter (**B**) in DEX-stimulated C2C12 myotubes. The data were based on triplicate experiments. Values are expressed as means ± SE. Values not sharing a common letter (a, b, c, d) are significantly different among groups. Images captured at ×200 magnification. CON: non-treated cells; DEX: dexamethasone.

We measured the viability of DEX-treated mature myotubes using the MTS assay. The DEX group (100 μM) showed significantly decreased cell viability (by 25.43%) compared to the non-treated cells (CON group). In contrast, TME-treated groups restored cell viability in a dose-dependent manner (Figure 4A). Morphological analysis showed that DEX treatment caused cell membrane shrinkage, whereas the TME treatment restored the DEX-induced reduction in myotube diameter (Figure 4B).

### 3.5. Effects of TME on Dexamethasone-Induced Muscle Atrophy-Related Gene Expression

**Figure 5:**
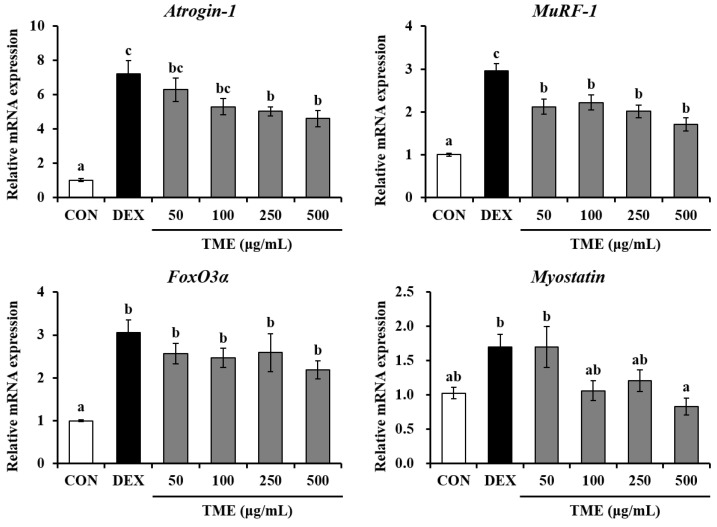
Effects of *Tenebrio molitor* larvae ethanol (TME) extract on mRNA expression of E3 ubiquitin ligases, FoxO3a, and myostatin in DEX-stimulated C2C12 myotubes. Glyceraldehyde-3-phosphate dehydrogenase (GAPDH) was used as an internal reference gene. The data were based on triplicate experiments. Values are expressed as means ± SE. Values not sharing a common letter (a, b, c) are significantly different among groups. CON: non-treated cells; DEX: dexamethasone; *MuRF-1*: muscle RING finger protein-1; *FoxO3α*: forkhead box O3.

To identify the inhibitory effects of the TME on DEX-induced muscle atrophy, we measured the gene expression of atrophic muscle markers. DEX stimulation caused a significant increase in the mRNA expression of *Atrogin-1*, *MuRF-1*, and forkhead box O3 (*FoxO3α*). In contrast, TME treatment markedly reduced *Atrogin-1* gene expression in a dose-dependent manner (Figure 5). TME at all tested doses (50–500 μg/mL) significantly downregulated the DEX-induced increase in *MuRF-1* expression. *FoxO3α* gene expression was reduced by TME treatment (50–500 μg/mL), although these changes were not statistically significant. The *myostatin* gene level was slightly decreased compared to that in the DEX group at 100 or 250 μg/mL TME treatment, and was significantly reduced at high concentrations (500 μg/mL). 

### 3.6. Effects of TME on Dexamethasone-Induced Muscle Atrophy-Related Protein Expression

**Figure 6:**
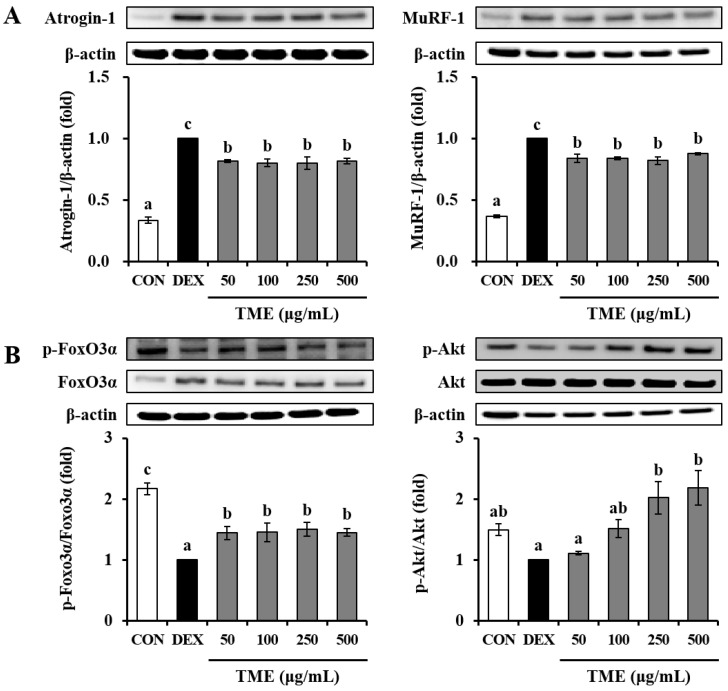
Effects of *Tenebrio molitor* larvae ethanol (TME) extract on protein expression of E3 ubiquitin ligases (**A**) and activation of FoxO3a and Akt in DEX-stimulated C2C12 myotubes (**B**). β-actin was used as an internal standard. The phosphorylation protein levels were normalized to levels of total proteins, and are presented as fold-change relative to the DEX group. The data were based on triplicate experiments. Values are expressed as means ± SE. Values not sharing a common letter (a, b, c) are significantly different among groups. CON: non-treated cells; DEX: dexamethasone; MuRF-1: muscle RING finger protein-1; FoxO3α: forkhead box O3; Akt: protein kinase B.

We further confirmed the protein expression of atrophic muscle markers. Cells treated with DEX exhibited a significant increase in Atrogin-1 and MuRF-1 protein levels compared to those in the untreated CON group. Atrogin-1 and MuRF-1 protein expression levels were suppressed by TME treatment (Figure 6A). The phosphorylation of FoxO3α was markedly downregulated in the DEX-treated group relative to the untreated CON group. In contrast, the TME at all tested doses (50–500 μg/mL) significantly up-regulated them (Figure 6B). Phosphorylation of protein kinase B (Akt) tended to be lower in the DEX group than in the CON group. However, TME treatment (250 and 500 μg/mL) significantly increased Akt phosphorylation compared to the DEX group. Thus, the TME protected C2C12 myotubes against DEX-induced muscle atrophy.

## 4. Discussion

In this study, we evaluated the beneficial effects of TME on muscle function through an increase in skeletal muscle hypertrophy and the recovery of DEX-induced muscle atrophy in C2C12 myotubes. Muscle development and differentiation are regulated by four transcription factors: myogenic regulatory factors (MRFs) [12]. These MRFs play different roles during myogenesis, with varying levels of expression. The primary function of MRFs is to specify the skeletal muscle lineage (Myf5 and MyoD), or to coordinate differentiation (MyoG), and MRF4 (Myf6) controls both aspects of these activities [12,13]. CK is an essential phosphoryl transferase enzyme involved in cellular energy homeostasis and is used as an indicator of differentiation [14]. In particular, MyoG transcript levels steadily increase during myoblast differentiation into myotubes [15,16], and MyoG knockdown has been shown to induce defective terminal muscle differentiation [15,17]. In this study, TME exerted a stimulatory effect on myoblast differentiation into myotubes by increasing the mRNA and protein expression of *MyoG*. TME also induced the gene expression of myogenic differentiation markers such as *Myf5*, *Myf6*, and *CKm*. In addition, TME treatment enhanced myotube formation, as indicated by the morphological analysis.

A previous study reported that phosphoinositide 3-kinase (PI3K) activity and mTOR are required for MyHC expression, myotube formation, and transcriptional activation of the *MyoG* and *Ckm* genes [18]. The mTOR signaling pathway is mediated by mTOR complex 1 (mTORC1) and mTOR complex 2 (mTORC2). mTORC1 regulates protein synthesis by activating S6 kinase 1 (S6K1) and inhibiting 4E-BP1 [8,19]. In contrast, mTORC2 phosphorylates AGC kinases, serum/glucocorticoid-regulated kinase 1 (SGK1), protein kinase C (PKC), and Akt, and regulates cell survival and metabolism [8,20]. Activation of mTOR induces the phosphorylation of 4E-BP1, which dissociates 4E-BP1 from the mRNA cap-binding protein eIF4E and promotes protein synthesis [21]. Therefore, we suggest that TME promotes protein synthesis by activating the mTORC1 signaling pathway in C2C12 myotubes.

The anti-inflammatory drug DEX, a synthetic glucocorticoid, can cause many side effects, such as muscle atrophy when administered in high-doses, or in long-term usage [22]. DEX-induced muscle atrophy is caused by increased muscle protein degradation and decreased synthesis [22]. Qin et al. [23] demonstrated that DEX-induced skeletal muscle atrophy is associated with the upregulation of myostatin promoter activity. In our results, *myostatin* gene expression was significantly decreased by TME treatment (500 μg/mL) compared with the DEX group. Among the FoxO isoforms in skeletal muscle, the FOXO3α is involved in the ubiquitin-proteasome system and the autophagy pathway. It activates the expression of E3 ubiquitin ligases such as atrogin-1/MAFbx [24]. DEX-induced myotube atrophy results in the dephosphorylation of Akt, leading to the activation of FoxO, and transcription of atrogin-1/MAFbx and MuRF1 [25]. A previous study reported that overexpression of MAFbx in myotubes caused atrophy, whereas mice deficient in either MAFbx or MuRF1 were resistant to atrophy [26]. These findings, combined with our results, demonstrate that the TME could protect against DEX-induced myotube atrophy by promoting the phosphorylation of Akt and FoxO3α.

Previous studies have shown that TME contains antioxidants such as phenolic compounds and flavonoids [27]. Bioactive peptides, including antimicrobial peptides, have been reported to have various biological effects [28]. Although components of TME were not determined in this study, it can be inferred that the antioxidants and bioactive peptides present in TME may be involved in its protective function against muscle atrophy.

## 5. Conclusions

In conclusion, the TME could enhance skeletal myotube hypertrophy by stimulating the mTOR signaling pathway. It was also shown to rescue DEX-induced muscle atrophy by alleviating atrophic muscle markers through phosphorylation of Akt and FoxO3α. Thus, TME has the potential to be a useful therapeutic agent for treating muscle weakness and atrophy. Future research should investigate muscle regeneration and anti-sarcopenic effects of TME in mouse skeletal muscle atrophy model to extend its utility as an alternative agent for treating sarcopenia.

## Figures and Tables

**Table 1:** Primer sequences for reverse transcription quantitative real-time PCR.

Gene	Full Name	Forward/Reverse(5′–3′)
*Atrogin-1*	F-box protein 32	TCATGCAGAGGCTGAGTGAC/ TCAAACGCTTGCGAATCTGC
*Ckm*	Muscle creatine kinase	CTTCCTGTTTGACAAGCCCG/ CTCCTCGTTCACCCACACAA
*Foxo3α*	Forkhead box O3	TGAAGGGAAGGAGCCGAGGTA/ GCTCTCTCCTCTCGAGCCCA
*Gapdh*	Glyceraldehyde-3-phosphate dehydrogenase	GGAGAGTGTTTCCTCGTCCC/ ATGAAGGGGTCGTTGATGGC
*MuRF-1*	Muscle RING finger protein-1	ACACATAGCAGAGGCCTTGAG/ TCTTTACCCTCTGTGGTCACG
*Myf5*	Myogenic factor 5	ACCCTAACCAGAGACTCCCC/ GTCCCGGCAGGCTGTAATAG
*Myf6*	Myogenic factor 6	CCACAGATCGTCGGAAAGCA/ CAGTCTCTGGTTGGGGTTGG
*MyoD*	Myogenic differentiation 1	TACAGTGGCGACTCAGATGC/ CACTGTAGTAGGCGGTGTCG
*MyoG*	Myogenin	CCCTACAGACGCCCACAATC/ AGTTGGGCATGGTTTCGTCT
*Myostatin*	Myostatin	TGCTGTAACCTTCCCAGGAC/ TCAAGCCCAAAGTCTCTCCG

## Data Availability

Not applicable.

## References

[1] Walston J.D. (2012). Sarcopenia in older adults. Curr. Opin. Rheumatol..

[2] Cohen S., Nathan J.A., Goldberg A.L. (2015). Muscle wasting in disease: Molecular mechanisms and promising therapies. Nat. Rev. Drug Discov..

[3] Son Y.H., Lee S.J., Lee K.B., Lee J.H., Jeong E.M., Chung S.G., Park S.C., Kim I.G. (2015). Dexamethasone downregulates caveolin-1 causing muscle atrophy via inhibited insulin signaling. J. Endocrinol..

[4] Hasselgren P. (1999). Glucocorticoids and muscle catabolism. Curr. Opin. Clin. Nutr. Metab. Care.

[5] Lecker S.H., Goldberg A.L., Mitch W.E. (2006). Protein degradation by the ubiquitin–proteasome pathway in normal and disease states. J. Am. Soc. Nephrol..

[6] Gumucio J.P., Mendias C.L. (2013). Atrogin-1, MuRF-1, and sarcopenia. Endocrine.

[7] Clarke B.A., Drujan D., Willis M.S., Murphy L.O., Corpina R.A., Burova E., Rakhilin S.V., Stitt T.N., Patterson C., Latres E. (2007). The E3 Ligase MuRF1 degrades myosin heavy chain protein in dexamethasone-treated skeletal muscle. Cell Metab..

[8] Yoon M.S. (2017). mTOR as a key regulator in maintaining skeletal muscle mass. Front. Physiol..

[9] Feng S. (2018). *Tenebrio molitor* L., entomophagy and processing into ready to use therapeutic ingredients: A review. J. Nutr. Health Food Eng..

[10] Kim Y., Yoon Y., Oh E. (2020). Effect on myogenesis and anti-inflammation of mealworm (*Tenebrio molitor*) larvae protein hydrolysate. Curr. Dev. Nutr..

[11] Lee J.B., Kwon D.K., Jeon Y.J., Song Y.J. (2021). Mealworm (*Tenebrio molitor*)-derived protein supplementation attenuates skeletal muscle atrophy in hindlimb casting immobilized rats. Chin. J. Physiol..

[12] Pajalunga D., Crescenzi M. (2021). Restoring the cell cycle and proliferation competence in terminally differentiated skeletal muscle myotubes. Cells.

[13] Berkes C.A., Tapscott S.J. (2005). MyoD and the transcriptional control of myogenesis. Semin. Cell Dev. Biol..

[14] Kim M., Sung B., Kang Y.J., Kim D.H., Lee Y., Hwang S.Y., Yoon J.H., Yoo M.A., Kim C.M., Chung H.Y. (2015). The combination of ursolic acid and leucine potentiates the differentiation of C2C12 murine myoblasts through the mTOR signaling pathway. Int. J. Mol. Med..

[15] Mielcarek M., Isalan M. (2021). Kinetin stimulates differentiation of C2C12 myoblasts. PLoS ONE.

[16] Andrés V., Walsh K. (1996). Myogenin expression, cell cycle withdrawal, and phenotypic differentiation are temporally separable events that precede cell fusion upon myogenesis. J. Cell Biol..

[17] Dedieu S., Mazeres G., Cottin P., Brustis J. (2002). Involvement of myogenic regulator factors during fusion in the cell line C2C12. Int. J. Dev. Biol..

[18] Sumitani S., Goya K., Testa J.R., Kouhara H., Kasayama S. (2002). Akt1 and Akt2 differently regulate muscle creatine kinase and myogenin gene transcription in insulin-induced differentiation of C2C12 myoblasts. Endocrinology.

[19] Ma X.M., Blenis J. (2009). Molecular mechanisms of mTOR-mediated translational control. Nat. Rev. Mol. Cell Biol..

[20] Sarbassov D.D., Ali S.M., Sabatini D.M. (2005). Growing roles for the mTOR pathway. Curr. Opin. Cell Biol..

[21] Yang H.Y., Xue L.Y., Xing L.X., Wang J., Wang J.L., Yan X., Zhang X.H. (2013). Putative role of the mTOR/4E-BP1 signaling pathway in the carcinogenesis and progression of gastric cardiac adenocarcinoma. Mol. Med. Rep..

[22] Jiang R., Wang M., Shi L., Zhou J., Ma R., Feng K., Chen X., Xu X., Li X., Li T. (2019). *Panax ginseng* total protein facilitates recovery from dexamethasone-induced muscle atrophy through the activation of glucose consumption in C2C12 myotubes. Biomed. Res. Int..

[23] Qin J., Du R., Yang Y., Zhang H., Li Q., Liu L., Guan H., Hou J., An X. (2013). Dexamethasone-induced skeletal muscle atrophy was associated with upregulation of myostatin promoter activity. Res. Vet. Sci..

[24] Clavel S., Siffroi-Fernandez S., Coldefy A.S., Boulukos K., Pisani D.F., Dérijard B. (2010). Regulation of the intracellular localization of Foxo3a by stress-activated protein kinase signaling pathways in skeletal muscle cells. Mol. Cell. Biol..

[25] Sandri M., Sandri C., Gilbert A., Skurk C., Calabria E., Picard A., Walsh K., Schiaffino S., Lecker S.H., Goldberg A.L. (2004). Foxo transcription factors induce the atrophy-related ubiquitin ligase atrogin-1 and cause skeletal muscle atrophy. Cell.

[26] Bodine S.C., Latres E., Baumhueter S., Lai V.K., Nunez L., Clarke B.A., Poueymirou W.T., Panaro F.J., Na E., Dharmarajan K. (2001). Identification of ubiquitin ligases required for skeletal muscle atrophy. Science.

[27] Jin K.N., Jeong E.J., Kim Y.S. (2021). Antioxidant components and antioxidant activities of mealworm (*Tenebrio molitor* larvae). J. Food Hyg. Saf..

[28] Errico S., Spagnoletta A., Verardi A., Moliterni S., Dimatteo S., Sangiorgio P. (2022). *Tenebrio molitor* as a source of interesting natural compounds, their recovery processes, biological effects, and safety aspects. Compr. Rev. Food Sci. Food Saf..

